# H3 histone methylation landscape in male urogenital cancers: from molecular mechanisms to epigenetic biomarkers and therapeutic targets

**DOI:** 10.3389/fcell.2023.1181764

**Published:** 2023-05-09

**Authors:** Liliana Burlibasa, Alina-Teodora Nicu, Mariana Carmen Chifiriuc, Cosmin Medar, Amelia Petrescu, Viorel Jinga, Ileana Stoica

**Affiliations:** ^1^ Faculty of Biology, University of Bucharest, Bucharest, Romania; ^2^ Academy of Romanian Scientists, Bucharest, Romania; ^3^ Romanian Academy, Bucharest, Romania; ^4^ University of Medicine and Pharmacy “Carol Davila”, Bucharest, Romania; ^5^ Clinical Hospital “Prof. dr Theodor Burghele”, Bucharest, Romania

**Keywords:** histone methylation, HMT, HDM, epidrugs, epigenetic biomarkers, genitourinary cancer

## Abstract

During the last decades, male urogenital cancers (including prostate, renal, bladder and testicular cancers) have become one of the most frequently encountered malignancies affecting all ages. While their great variety has promoted the development of various diagnosis, treatment and monitoring strategies, some aspects such as the common involvement of epigenetic mechanisms are still not elucidated. Epigenetic processes have come into the spotlight in the past years as important players in the initiation and progression of tumors, leading to a plethora of studies highlighting their potential as biomarkers for diagnosis, staging, prognosis, and even as therapeutic targets. Thus, fostering research on the various epigenetic mechanisms and their roles in cancer remains a priority for the scientific community. This review focuses on one of the main epigenetic mechanisms, namely, the methylation of the histone H3 at various sites and its involvement in male urogenital cancers. This histone modification presents a great interest due to its modulatory effect on gene expression, leading either to activation (e.g., H3K4me3, H3K36me3) or repression (e.g., H3K27me3, H3K9me3). In the last few years, growing evidence has demonstrated the aberrant expression of enzymes that methylate/demethylate histone H3 in cancer and inflammatory diseases, that might contribute to the initiation and progression of such disorders. We highlight how these particular epigenetic modifications are emerging as potential diagnostic and prognostic biomarkers or targets for the treatment of urogenital cancers.

## 1 Introduction

Genitourinary cancers represent 13% of all cancers, being responsible for a cumulative number of 776.524 deaths worldwide in 2020, which is expected to grow in the future. This pathology can occur in all age groups, with some being more prevalent in young adults and others in elders (Global Cancer Observatory). The main types of genitourinary cancers are, in decreasing order of their frequency: prostate, bladder, kidney, and testicular cancer. Although prostate cancer (PCa) is one of the leading causes of death in men of any age, testicular cancer (TCa) is the most commonly diagnosed type in young men and poses a real threat to male fertility (Van Hemelrijck et al., 2013; Hamano et al., 2017). While these two subtypes are exclusive to men, kidney and bladder cancer can affect both sexes. However, they are more common in men, with bladder cancer being the 10th most common cancer worldwide, and kidney cancer, particularly the renal cell carcinoma (RCC) subtype, being the deadliest in the group (Global Cancer Observatory).

Although the genetic and environmental risk factors are different for various types, certain risk factors such as cigarette smoking and older age are common (O’Rourke et al., 2013). With the increasing interest in epigenetics over the past decades, some light has been shed on the epigenome alteration by environmental factors and the involvement of epigenetic mechanisms in genitourinary cancers.

The field of epigenetics, albeit relatively new compared to genetics, has boomed over the last 2 decades with more and more studies focusing on the thorough investigation of certain mechanisms or on the epigenomics landscape. Traditionally, epigenetics refers to inheritable modifications that alter gene expression but not the DNA sequence (Waterland, 2006). Different definitions disregard the inheritable aspect, including long-term alterations that are not necessarily inherited (Bird, 2007). These modifications concern several mechanisms, namely, DNA methylation, chromatin remodeling, RNA-based mechanisms, histone variants and post-translational modifications (PTMs) of histones. Historically, DNA methylation was the first epigenetic mechanism to be investigated and has remained to this day the most studied, including in relation to cancer. RNA-based mechanisms are also popular choices these days, particularly microRNAs which are easily detected in bodily fluids, but can be unstable. PTMs of histones play an essential role in gene regulation and often crosstalk with DNA methylation and chromatin remodeling but they are studied to a lesser extent. There are numerous types of PTMs, including acetylation, methylation, phosphorylation, ubiquitination and sumoylation, each having its own role in gene regulation as well as in other mechanisms, like DNA repair, cell division and differentiation (Burlibaș;a and Zărnescu, 2013; Gong and Miller, 2019; Aquila and Atanassov, 2020). In cancer, histone acetylation is the most studied PTM, as its regulator enzymes, histone-acetyl and -deacetyl transferases (HATs/HDACs) have been associated with tumorigenesis. HAT genes can be considered oncogenes or tumor suppressor genes depending on a particular context, while HDAC inhibitors have already been approved by the Food and Drug Administration (FDA) for the treatment of certain cancers (e.g., belinostat) (Wu et al., 2020). This interest does not fully transfer to histone methylation yet, but progress has been made in this direction.

**FIGURE 1:**
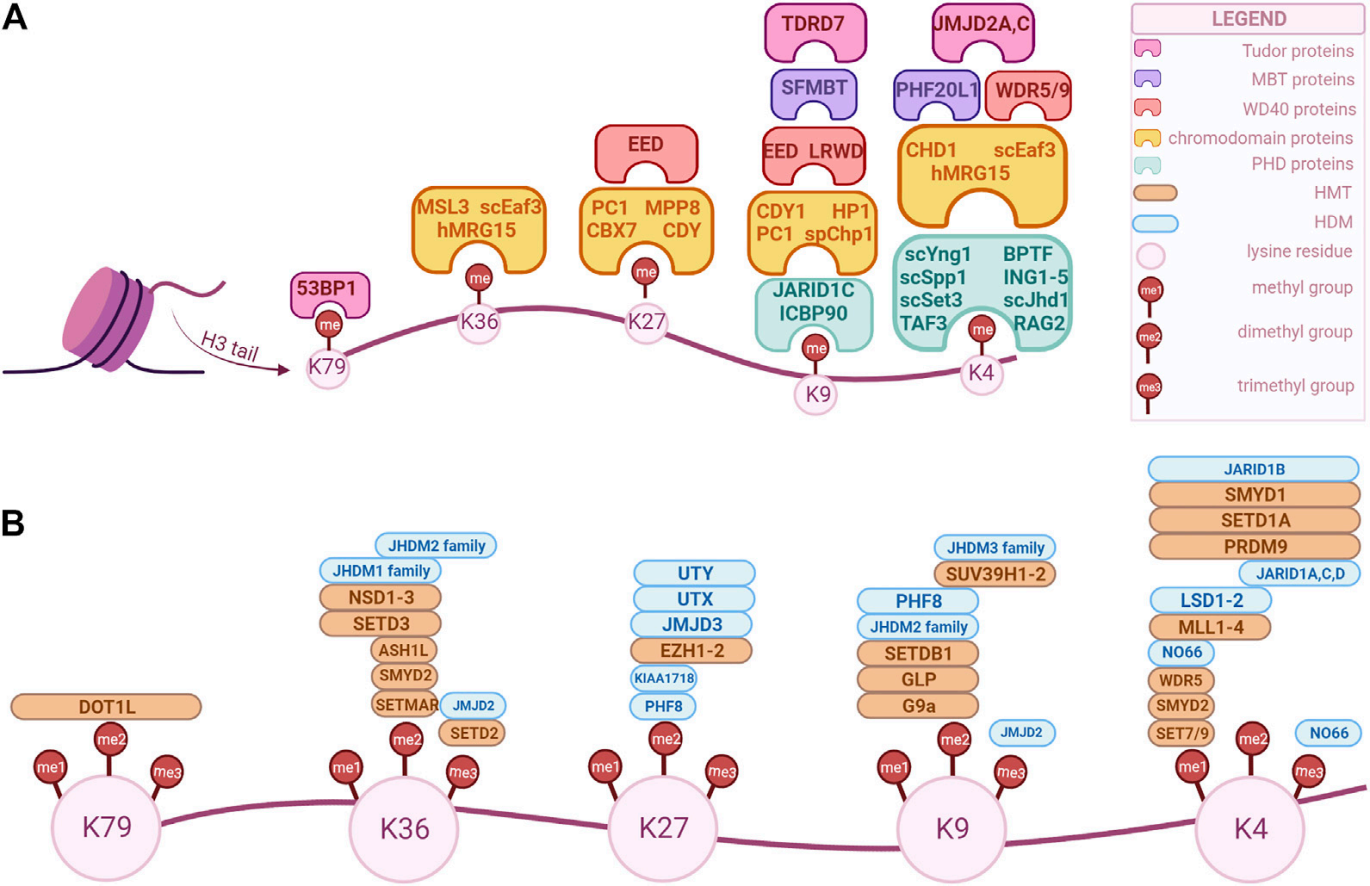
Overview of H3 key modifiers that modulate and/or recognize epigenetic marks in human cells. These factors can be classified as “writers”, “readers” and “erasers” based on their biochemical activity. The epigenetic marks tailored by these factors are currently being explored as potential biomarkers for diagnosis, prognosis and treatment option for genitourinary cancers. **(A)** “Readers” with specific domains of interaction. **(B)** “Writers” and “erasers” that modulate methylation of specific lysine position in H3. The enzymes’ positions correspond to their specificity, i.e., an enzyme positioned over a “me1” but not over “me2” is specific only for that methylation state, while an enzyme that is positioned over both can modulate those methylation states. (Created with BioRender.com).

Among the five canonical histone types, methylation is the most common in histone H3. Lysine or arginine residues can be methylated by histone methyltransferases (HMTs) and demethylated by histone demethylases (HDMs). Depending on the amino acid residue, its methylation can be associated with either activation or repression of transcriptional activity. Chromatin remodeling complexes can also interact with methylated residues and their corresponding enzymes, affecting the nucleosomal structure and, thus, regulating transcriptional activity as well (Cardoso et al., 2021). Moreover, methylated histones extend their role to DNA repair, replication, and cell-cycle control (Allis and Jenuwein, 2016). Given that there are three states of methylation (mono-, di- and tri-methylation), multiple residues that can be methylated and several enzymes that could catalyze the same residue (Cardoso et al., 2021)., there is a vast array of HMTs and HDMs and several instances of crosstalk between different modifications, which are depicted in Figure 1. In the next sections, we will give a brief description of the main modifications and their regulators, followed by the thorough analysis of each of them in relation to the genitourinary cancers. This review aims to provide an update on the current literature concerning H3 methylation landscape in genitourinary cancers, highlighting its importance in cancer initiation, differentiation, and progression. Moreover, areas of interest for future research are identified, with a focus on new biomarkers development and tailoring the epigenome through therapeutic approaches.

## 2 H3 methylation patterns and regulators

Histone H3 has multiple lysine and arginine residues that can be methylated. To date, the known lysine sites are H3K4/9/14/18/23/27/36/37/56/64/79/112, and the known arginine sites are H3R2/8/17/26/42 (Yang et al., 2022). While all of these sites can be methylated under certain biological conditions, only some of them have been associated with cancer. As such, in the following sections we will focus only on the later, particularly in regards to urogenital cancers.

Histone 3-lysine4 (H3K4) methylation is associated with gene activation and can usually be found at promoter regions and transcriptional start sites (Shilatifard, 2012). The state of methylation is associated with different localizations, H3K4me1 being enriched at enhancers, H3K4me2 at the 5’ end of transcribing genes, and H3K4me3 at promoters of active or poised genes (Hyun et al., 2017). The SET7/9 enzyme (Su(var)-3-9, Enhancer-of-Zeste, Trithorax, domain containing protein 7/9), also referred to as SET7, SETD7, SET9, or KMT7, is a SET domain-containing lysine methyltransferase that mono-methylates H3K4 and activates gene transcription (Xiao et al., 2003; Gu et al., 2022). Additional methylations can be achieved by MLL proteins, but the mechanism by which SET/MLL complexes can differentially methylate H3K4 is not known (Hyun et al., 2017). Demethylation, initially thought to be stable, is catalyzed by Lysine specific demethylase 1 (LSD1/KDM1A) which demethylates mono- or di-methylated histone lysine 4 (H3K4me1,2 - enhancer-associated histone marks), and interacts with histone deacetylases 1, 2 (HDAC1,2). Moreover, LSD1 is recruited to target genes by repressive complexes, for instance CoREST. Additional H3K4 demethylases exist, such as KDM5A/B/C/D and MAPJD (Højfeldt et al., 2013). As previously mentioned, histone modifications can interact with other mechanisms through being recognized by effectors known as “readers”. Chromatin remodeling enzymes, such as CHD1 and the NURF (Nucleosome Remodeling Factor) complex can be recruited by H3K4me2 and H3K4me3, respectively (Hyun et al., 2017). Mutations of either “readers”, “writers” (HMTs) or “erasers” (HDMs) have been associated with different types of cancers.

Methylation of lysine 9 (H3K9) and lysine 27 (H3K27) in histone 3 are repressive modifications that are likely to preserve heterochromatin stability (Allis and Jenuwein, 2016). EHMT1 (euchromatic histone methyltransferase 1) methylates H3K9, while G9a, a histone methyltransferase, has the capacity to di-methylate H3K9. Trimethylation can be achieved by SUV39H1/2. JMJD2A, also named lysine-specific demethylase 4A (KDM4A), is a demethylase that belongs to the family of Jumonji domain proteins (JMJD), with the main function of demethylating H3K9me3 (Kooistra and Helin, 2012). H3K9 methylation has been shown to interact with DNA methylation, as they are both involved in heterochromatin formation and DNA methylation maintenance during replication. This is explained by the fact that SUV39H1/2 and DNMT3A/B are recruited by HP1 and can interact with each other, while G9a and DNMT1 colocalize with PCNA at the replication fork (Rose and Klose, 2014; Hyun et al., 2017).

H3K27me3 is regulated by Enhancer of Zeste homolog 2 (EZH2), an enzymatic catalytic subunit of polycomb repressive complex 2 (PRC2). The main functional domains of EZH2 are cysteine-rich region and SET domain (Hamamoto and Nakamura, 2016). Also, EZH2 requires two other polycomb subunits, EED and SUZ12, to function as a histone methyltransferase (Kim and Roberts, 2016). Demethylation is achieved by KDM6A/B/C, which are thus associated with derepression of genes. KDM6, also known as UTX is a subunit in the MLL4 H3K4 methyltransferase complex and can mediate crosstalk between H3K4 and H3K27. H3K27me3 is mainly involved in the maintenance of gene repression, being commonly associated with the inactivation of chromosome X in mammalian females (Hyun et al., 2017).

H3K36 methylation can regulate transcription initiation, elongation, DNA repair and alternative splicing. NSD2 (nuclear receptor binding SET domain protein 2), also named MMSET (multiple myeloma SET domain), catalyzes H3K36me2 formation and has been associated with cancer (Ezponda et al., 2013). Additionally, there are seven other HMTs and two HDMs, of which JmjC-domain containing proteins (JHDM1) are responsible for distributing H3K4me3 and H3K36me3 in different genes (Bannister and Kouzarides, 2011). Furthermore, H3K36me3 is an antagonist of the PRC2 complex and as such it cannot colocalize with H3K27me3 (Yuan et al., 2011).

H3K79 methylation is a histone modification driven by the disruptor of telomeric silencing 1 (DOT1L) methyltransferase (Martin and Zhang, 2005). Unlike the previous residues, H3K79 is part of the globular domain of H3. It plays a role in gene activation, both in mono- and demethylated states, and its “writer” has long been associated with leukemia (Hamamoto and Nakamura, 2016).

**FIGURE 2:**
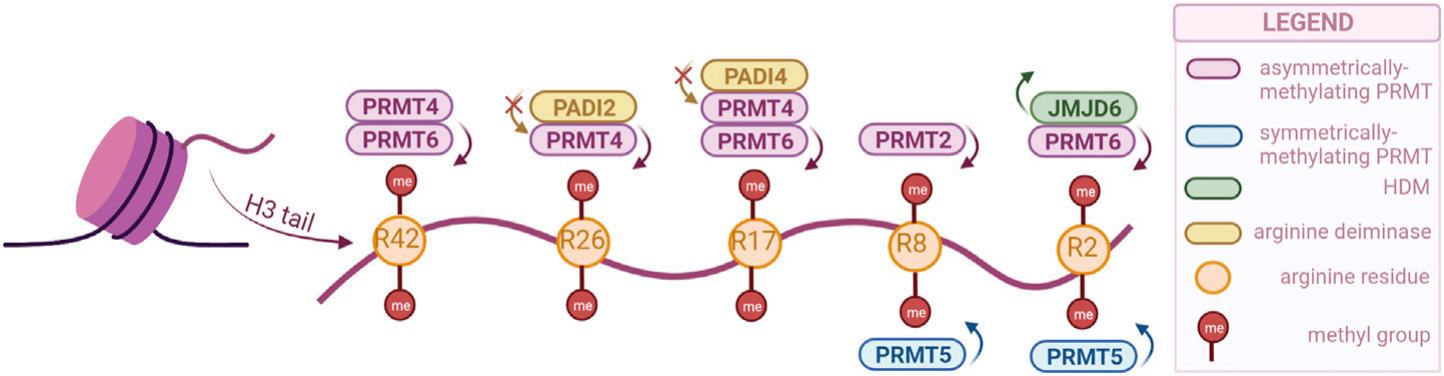
PRMTs and the sites they preferentially methylate on the H3 tail, as well as the arginine-specific HDMs. PRMT2,4 and 6 catalyze the asymmetrical dimethylation of arginine residues, while PRMT5 catalyzes symmetrical dimethylation (PRMTs with other specificities not illustrated). Demethylation can only be catalyzed by JMJD6, but methylation can also be prevented by PADI proteins, through deimination (citrullination) of arginines. (Created with BioRender.com).

Arginine methylation has been studied less than lysine methylation, and as such not that much is known about the function of the different sites. However, the enzymes responsible for this modification have been gathering more attention in the past years as they have been associated with crucial biological processes and even with cancer. These enzymes are Protein Arginine Methyltransferases (PRMTs), and they are divided into three classes: type 1, responsible for asymmetric demethylation; type 2, responsible for symmetric di-methylation; and type 3, responsible for mono-methylation (Liu et al., 2020). Their activity is not limited to histones, as they can methylate other proteins as well. There are nine PRMTs (PRMT1-9) in mammalian cells, of which PRMT1 is the most prominent. Brief descriptions of each PRMT and their various functions can be found in reviews by Di Lorenzo and Bedford (2011); Guccione and Richard (2019), while our review will only highlight the enzymes associated with urogenital cancers (Figure 2).

Similar to lysine residues, some marks have been associated with gene activation, namely, H3R2me2s, H3R17me2a, H3R26me2a, and others with repression, H3R2me2a, H3R8me2a, H3R8me2s. Notably, methylation symmetry status is important for the function. Besides regulating the expression of tumor-related genes, PRMTs are involved in tumorigenesis through other processes as well, such as mRNA splicing and DNA damage response (Dai et al., 2022).

Arginine demethylation is catalyzed by JMJD6 and peptidylarginine deiminases (PADIs). JMJD6 was the first to be identified, and has also been the most studied, as there is a clear connection with tumorigenesis. A recent study has even used a JMJD6 inhibitor to show its anti-tumorigenesis potential both *in vivo* and *in vitro* (Xiao et al., 2022). While JMJD6 can demethylate H3R2me2, PADIs are not necessarily able to demethylate, but they can block methylation by converting the arginine residue to citrulline (Di Lorenzo and Bedford, 2011).

## 3 Prostate cancer (PCa)

Prostate cancer is one of the most commonly diagnosed oncological conditions, with estimated 1,414,000 (incidence 14.1%) new cancer cases and 375,304 deaths reported in 2020 (Wang et al., 2022). Many risk factors have been identified, but the exact causes of PCa have yet to be discovered. Perturbed transcriptional control is one of the main mechanisms that promote both prostate cancer differentiation and proliferation. In recent years, multiple molecular key players for PCa development have been identified, such as androgen receptor (AR), IDH1, FOXA1, TP53, RB1, PTEN, N-myc, ETS family transcription factors, OCT4, SOX2, NANOG, etc. (Robinson et al., 2015; Amin et al., 2017).

The prostate tumors have a high level of heterogeneity suggesting that there could be several events leading to inactivation of some tumor suppressor genes or to activation of oncogenes that could, at some point of disease progression, interfere with AR (Boyd et al., 2012; The Cancer Genome Atlas Research Network, 2014). In this regard, a well-studied pathway is the oncogenic activation of the phosphatidylinositol-3-kinase (PI3K), protein kinase B (PKB/AKT) and mammalian target of rapamycin (mTOR) which interact with AR promoting PCa development and impact the response to PI3K-AT-mTOR-targeted therapies (de Bono et al., 2019).

The androgen receptor is the most important transcription factor involved in the progression of PCa. As such, for advanced PCa treatment, androgen deprivation (ADT) is currently part of the gold standard therapy (Kawakami et al., 2006). Even though ADT has doubtlessly improved survival for PCa patients, it is not curative in all cases. Relapse occurs in about 30% of cases, which progress to castration-resistant prostate tumors (CRPC), associated with gene silencing resulting from deregulated epigenetic modifications, such as DNA and histone methylation (Ruggero et al., 2018; Moreira-Silva et al., 2022).

Some AR inhibitors, such as enzalutamide and abiraterone (CYP17A1 inhibitor), have been approved by the FDA for the treatment of advanced stage PCa (Rodriguez-Vida et al., 2015). However, some cases develop resistance to enzalutamide and abiraterone, making the disease almost incurable (Wang and Liu, 2020; Fu et al., 2023). Unfortunately, in most cases AR becomes again active, and these patients relapse (Sowalsky et al., 2015). Identifying the mechanism that drives these advanced tumors is still a challenge. Therefore, bypassing resistance to enzalutamide is currently under intensive research and new targets and mechanism-based strategies need to be developed in order to treat CRPC patients. A highly aggressive variant of CRPC is neuroendocrine prostate cancer (NEPC), which seems resistant to any current therapies for advanced PCa. NEPC is a small cell PCa with a very quick proliferation, which tends to metastasize to visceral organs (liver and lung), in contrast to CRPC which produces bone metastases (Chen J. et al., 2022).

The sequencing efforts for thousands of tumor specimens from patients with different stages of prostate cancer have highlighted a landscape of the genetic alterations that accompany the progression of this type of cancer (Ruggero et al., 2018). Together with these genetic drivers of cancer development, an important number of new alterations, especially key players in chromatin architecture and epigenetic regulators have been identified.

In what follows, we will highlight the role played by methylation/demethylation of H3 histone in the development and metastasis of PCa. Moreover, we will discuss the identification of new diagnostic biomarkers, focusing on H3 methylation modulators that are emerging as potential targets for anticancer therapy.

### 3.1 H3 methylation regulators in PCa

Due to the important role of PI3K/AKT signaling in cell proliferation, it is plausible that its activation crosstalks with histone modifications. A few studies have shown that PI3K pathway is involved in histone modification at both global level and gene-specific locus (Chen et al., 2008; Zuo et al., 2011).

In PCa cells, LSD1 activates the PI3K/AKT pathway in the absence of androgen stimulation and regulates the expression of P85, a PI3K subunit, through enhancer’s epigenetic reprogramming. Moreover, LSD1 could have a dual role in promoting PCa initiation by enhancing AR signaling via its coactivator function, and by activating PI3K/AKT pathway through increasing P85 gene expression (Wang et al., 2019). The overexpression of LSD1 was correlated with prostate cells’ malignancy, poor survival and recurrence of PCa (Kahl et al., 2006).

Research performed by Pang and his collaborators (2017) highlighted the role of PI3K/p110beta signaling in modulating androgen-stimulated H3K4me2 methylation in PCa cells, by controlling the intracellular localization of lysine-histone demethylase KDM5A. In the same study, scientists provided data linking p100beta specific inhibitor TGX221-induced tumor suppression with H3K4me2 (Pang et al., 2017). According to their findings, the level of H3K4 methylation is increased in CRPC compared to benign prostate tissues and primary tumors, uncovering a possible mechanism of p110beta-dependent modulation of H3K4me2 methylation during tumor progression. The mentioned study also identified H3K4me2 as a potential biomarker for prostate cancer prognosis (Pang et al., 2017). Another recent study has shown that AKT inhibition decreases the expression of the KDM5 family, especially KDM5B (JARID1B) in PTEN-null prostate cancer. AKT interferes with miR-137 resulting in increased levels of H3K4me2/3 as a consequence of transcriptionally repressed KDM5B. These results suggest that AKT-driven KDM5B expression is a key factor for tumorigenicity of PTEN PCa cells, identifying a mechanism by which AKT modifies the epigenome of tumoral cells, increasing the level of H3K4 methylation (Khan et al., 2019). Taken together these findings can be a starting point for the future development of combinatory therapies using epigenetic modulators and PI3K/AKT inhibitors in the PTEN-null PCa treatment. KDM5C (JARID1C), a H3K4me2/3–demethylase, is overexpressed in PCa, being associated with a reduced prostate-specific antigen survival (Stein et al., 2014).

Based on the findings that demonstrated high levels of H3K4 methylation, especially in metastatic CRPC (m-CRPC), many studies have focused on the dynamics of H3K4 methyltransferases. The MLL1 complex belongs to the KMT2/MLL family. MLL1 has a menin subunit through which it binds directly to the AR and acts as a co-activator of AR signaling (Brzezinka et al., 2020). Menin is a tumor suppressor encoded by the MEN1 gene that is over expressed in CRPC, being correlated with low overall survival in patients with prostate cancer (Malik et al., 2015). MLL2 activates the PI3K/EMT process and induces DNA damages in PCa (Lv et al., 2018; Yang et al., 2021). Another H3K4 methyltransferase found to have implications in prostate cancer is SMYD3. Its depletion inhibits PCa development by blocking the transcription of AR or cyclin D2 (Vieira et al., 2015). SMYD3 has dimethyl- and trimethyltransferase activity at lysine 4 of histone H3, but also methylates H4K5 and H4K20 and other non-histone proteins (Foreman et al., 2011). SMYD3 induces transcriptional activation of AR (Vieira et al., 2015) and as a consequence, PCa tissues displayed higher SMYD3 levels compared to normal prostate in advanced stages of disease (Vieira et al., 2014). In this case, the level of SMYD3 transcript conveys prognostically relevant information which could discriminate among different aggressiveness types of PCa.

SET7/9 preferentially monomethylates some non-histone proteins which participate in important cellular processes, including P53 (Kurash, et al., 2008), hypoxia-inducible factor 1α (HIF1α) (Liu et al., 2015), E2F1 transcription factors family (Irwin et al., 2000), FoxO3 (Li et al., 2019), Gli1/3 (a zinc finger transcription factor) (Fu et al., 2016), DNA cytosine methyltransferase 1 (DNMT1) (Estève et al., 2009), estrogen receptor (ER) and AR (Mellado et al., 2013), etc. This enzyme enhances AR transcriptional activity by methylating its K632 residue (Wang et al., 2018). SET7/9 has a proliferative role in prostate cancer, being involved in PTEN/PI3K/AKT signaling (Sowalsky et al., 2015; Yang et al., 2021).

Another enzyme which is present in high levels in m-CRPC is SETD1A. It trimethylates H3K4 and binds to E2F1 in the promoter region of the FOXM1 gene. SETD1A is also important for the expression of OCT4 and contributes to the proliferation of prostate stem cells, an event involved in m-CRPC. SETD1A-FOXM1 overexpression is correlated with a poor prognosis, suggesting that it can be used as an important marker for predicting the prognosis of m-CRPC (Yang et al., 2020).

A recent study revealed that H3K9me3 is essential for heterochromatin maintenance and progression to anti-androgen resistance in PCa. Increased expression of EHMT1 that methylates H3K9 was associated with poor patient hormonal therapy outcomes. Also, elevated expression of SUV39H1/SUV39H2 confers resistance, whereas inhibiting H3K9me3 writers blocks anti-androgen resistance. These findings reveal a drug resistance network that integrates multiple signaling elements and highlight potential pharmacologic vulnerabilities (Baratchian et al., 2022). By inhibiting this epigenetic network, drug-resistant tumors can be re-sensitized and could elicit cytotoxic interferon responses.

G9a, along with the repressive proprieties on gene activity, also functions as a coactivator of AR (Jones et al., 2021), by interacting with HKX3.1 homeobox gene which contributes to prostate differentiation (Dutta et al., 2016), and is a coactivator for PSA induction (Chen et al., 2012). All these findings suggest that dysregulation of G9a may contribute to the initiation and progression of PCa, and the inhibition of G9a could enhance cancer treatment, making it a potential therapeutic target. Overexpression of G9a is associated with worse prognosis in patients with PCa (Motolani et al., 2021).

Even though in the scientific literature there is a huge volume of studies regarding epigenetic regulators involved in prostate tumor development, the role of histone demethylation, and the corresponding enzymes have remained understudied. A study performed by Kim and others (2016) revealed a functional interaction between JMJD2A (KDM4A) and DNA binding transcription factor ETV1 leading to overexpression of yes associated protein 1 (YAP1). This protein is a Hippo pathway component that has been associated with PCa aggressiveness. ETV1 facilitates the recruitment of JMJD2A to YAP1 promoter and as a result, changes in histone methylation occur in PCa. These results highlight a JMJD2A-ETV1-YAP1 crosstalk associated with PCa initiation, and thus, it could represent a suitable target for therapeutic management of PCa patients (Kim et al., 2016).

Trimethylation of Lys-27 in histone 3 (H3K27me3) is correlated with the inhibition of gene transcription. Ngollo and collaborators investigated genome-wide H3K27me3 histone methylation distribution using chromatin immunoprecipitation (ChIP) and promoter microarrays to identify differentially enriched regions in biopsy samples from PCa patients (Ngollo et al., 2017). This study identified an average of 386 H3K27me3 enriched genes on promoter regions in control group (healthy persons) versus 545 genes in Gleason Score (GS) ≤ 7 and 748 genes in GS > 7 groups. Using factorial discriminant analysis and ANOVA tools to compare H3K27me3 enriched-genes in prostate tumor biopsies and normal biopsies, a significant number of differentially-enriched genes were identified (NPHP3-AS1, MYO1D, ALG5, CBX1, GRID2, ING3, MSH6, FBXO11, GRIN3B, SPATS2, EXOSC8, SND1, TENM4 and TRA2A. According to the mentioned study, the genes listed above are potentially associated with prostate cancer, and H3K27me3 could emerge as a biomarker in poor-prognosis PCa (Ngollo et al., 2017).

EZH2, which catalyzes H3K27me3 formation, can also act independently from PRC2 and histone activities to activate the androgen receptor and cause CRPC (Simon and Lange, 2008). To date, functions of EZH2 in cells proliferation, senescence, apoptosis and genomic imprinting have been well established (Duan et al., 2020), but the concern about its roles in the pathophysiology of cancer is just starting. It seems that EZH2 induces anchorage-independent colony formation and cell invasion (Thienger and Rubin, 2020). Intriguing results have uncovered the dual role of EZH2 that can act as a transcriptional suppressor or as a coactivator of transcription depending on histone methylation status (Puzyrenko et al., 2022). Upon demethylation, H3K27 undergoes acetylation (H3K27ac), a histone modification associated with an opened chromatin state, which activates target oncogenes expression, a key metastasis facilitator being targeted by ongoing trials, as with palbociclib (Palmbos et al., 2021). It was found that the expression of EZH2 is elevated in the small-cell neuroendocrine PCa (NEPC) phenotype (Abida et al., 2019). The number of NEPC cases with enriched levels of EZH2 is still unclear and the mechanisms that regulate EZH2 expression in CRPC also remain elusive. JMJD3 (KDM6B) is a H3K27 di- and tri-demethylase, upregulated in different PCa tissues, but its involvement in PCa is still unclear (Xiang et al., 2007).

Methylation at H3K36 refers to di-methylation or tri-methylation, the former being catalyzed by NSD2 which has a role in PCa by modulating Twist family bHLH transcription factor 1 (TWIST1) to promote epithelial to mesenchymal transition and invasiveness in PCa cell lines (Ezponda et al., 2013). Very few studies have focused on the analysis of H3K36me3 in PCa, but their results suggest that H3K36me3 antagonizes the PRC2-mediated H3K27 methylation (Thienger and Rubin, 2020; Yuan et al., 2020). SETD2 is a histone methyltransferase that mediates H3K36 trimethylation. SETD2 downregulated expression has been found in advanced PCa, emphasizing its role in disease progression (Beltran et al., 2016). In a study performed by Yuan and others by assessing the tumor-suppressive function of SETD2 in PCa, it was clearly demonstrated that SETD2 monomethylates EZH2 at its 735 lysine residue, triggering a Smurf2 E3 ligase-dependent degradation (Yuan et al., 2020). Moreover, SETD2 missense mutation at the R1523 residue abrogates the interaction with EZH2 without affecting the H3K36me3 activity, concluding the possibility that modification of EZH2, rather than deceased H3K36 methylation, may be the oncogenic promoter. Also, SETD2 loss cooperates with PTEN loss to produce CRPC (Yuan et al., 2020). However, a small minority of CRPC patients (1.6%) had SETD2 mutations, thus the mechanism which contributes to the impairment of EZH2 is still unelucidated. A SETD2-AMPK-EZH2 functional axis in PCa was identified by Yuan and collaborators (2020). According to the mentioned researchers, AMP-dependent kinase (AMPK) activates FOXO3, a forkhead transcription factor, and regulates SETD2 expression. Using an AMPK antagonist, metformin, the PCa cell growth was inhibited in androgen-sensitive and castration-resistant tumors. Methylation of EZH2–K735 could be triggered by metformin which leads to reducing cell proliferation in CRPC and tumor growth. Metformin treatment causes an increase in the SETD2 level, while EZH2 levels decrease (Yuan et al., 2020).

Lastly, DOT1L methylates H3K79 and participates in the regulation of development, differentiation and proliferation of normal cells, being essential for maintaining enhancer-promoter interaction (Martin and Zhang, 2005). A recent study has shown that DOT1L is upregulated and is associated with a poor outcome of PCa (Vatapalli et al., 2020). Inhibition of DOT1L impaired the viability of AR positive (AR+) PCa cells, including CRPC and enzalutamide-resistant cells, thus DOTL1 is a potential therapeutic target for PCa.

Of the PRMTs involved in methylation of H3R, only PRMT4, PRMT5 and PRMT6 have been associated with the progression of prostate cancer. PRMT4 is a coactivator-associated arginine methyltransferase 1 (CARM1), functioning as a coactivator of androgen receptor (AR)-mediated signaling. It has been found that overexpression of CARM1 is associated with the development of prostate cancer (PCa) and its progression to androgen-independent PCa (Kim et al., 2010). Thus, CARM1 may represent a potential target in PCa therapeutic management. PRMT5 crosstalks with BRG1-and hBRM-based hSWI/SNF chromatin remodelers and induces symmetrical dimethylation of H3R8 (H3R8me2s), inhibiting the tumor suppressor genes. Dysregulation of PRMT5 has been observed in various types of cancer, including prostate (Hwang et al., 2021). PRMT6 causes asymmetric dimethylation in histone 3 at arginine 2, arginine 17, arginine 42 (H3R2me2a, H3R17me2a, and H3R42me2a) (Cheng et al., 2020). H3R2me2a could have either inhibitory or activating properties depending on its genomic localization. Therefore, PRMT6 can enhance or inhibit cancer development. PRMT6 was found to be overexpressed in prostate tumor tissue associated with carcinogenic effect and poorer survival of patients (Vieira et al., 2014). Study performed by Almeida-Rios and others demonstrated that stable PRMT6 knockdown attenuated the malignant phenotype in PC-3 cell line (Almeida-Rios et al., 2016). Silencing of PRMT6 was linked to low H3R2me2a levels and high expression of MLL and SMTD3. Also, PRMT6 silencing increases p21, p27 and CD44 expression and decreases the level of MMP-9 which interferes with PI3K/AKT/mTOR and AR signaling pathway (Chen Z. et al., 2022). By restoring AR expression in Sh-PRMT6 PC-3, the inhibition of PRMT6 may restore sensitivity of androgen resistant cells, and could represent a particular approach for the CRPC treatment.

JMJD6 increases significantly with the progression of castration-resistant disease (Paschalis et al., 2021). As mentioned above, an important role in CRPC is played by AR, more specifically, a splice variant AR-7V. A strategy to avoid AR-V7 mediated resistance is to target epigenetic modulators involved in AR-V7 generation. JMJD6 has been reported to interact with proteins involved in RNA processing, for example, U2AF65 with critical role in expression of AR-V7 (Islam et al., 2019). Paschalis and others propose a hypothesis to explain CRPC progression using dual function enzyme JMJD6 and its JMJD6/U2AF65/AR-V7 axis. In this potential mechanism JMJD6 acts on AR promoter to demethylate H3R and promotes AR-V7 transcription. Knockdown of JMJD6 reduced cancer cells, AR-V7 levels and U2AF65 recruitment to AR pre-mRNA (Paschalis et al., 2021). These results demonstrated the association between JMJD6 and AR-V7 in CRPC and could be a potential therapeutic target.

PADI2 is H3 arginine demethylase, a key mediator for AR in prostate cancer progression (Wang et al., 2017; Zhu et al., 2022). This enzyme is able to alter gene transcription through citrullinating residues in H3. PADI2 promotes protein stability and transcriptional activation of AR in prostate cancer cell line. Consecutive demethylation with citrullination of H3R26 contributes to AR signaling activation and progression of CRPC (Wang et al., 2017).

The main H3 methylation key regulators considered as potential therapeutic targets are summarized in Table 1.

**TABLE 1:** Summary of H3 methylation master regulators considered as potential therapeutic targets in PCa.

Enzyme type	Enzyme name	PTMs	Function in PCa	References
HMTs	SMYD3	H3K4me2/3	Promotes cell proliferation and migration	Vieira et al. (2015)
	SUV39H1	H3K9me3	Enhance prostate cancer cell and migration	Baratchian et al. (2022)
	G9a	H3K9me2	Contributes to the development of PCa	Chen et al. (2012)
				Jones et al. (2021)
	EZH2	H3K27me2/3	Crucial key driver of prostate cancer development	Yang and Yu (2013)
				Abida et al. (2019)
	NSD2	H3K36me2	Promotes prostate cancer tumorigenesis and progression.	Ezponda et al. (2013)
	DOT1L	H3K79me2/3	Is necessary for the viability of AR^+^ PCa	Vatapalli et al. (2020)
	PRMT4 (CARM1)	H3R17me2a	Overexpressed in prostate cancer. Associated with tumorigenesis and cancer progression	Hong et al. (2004)
	PRMT5	H3R8me2a	Promotes development of CRPC	Beketova et al. (2022)
	PRMT6	H3R2/17/42me2a	Overexpressed in prostate cancer	Vieira et al. (2014)
HDMs	KDM5B (JARID1B)	H3K4me1/2/3	AR coactivator. Upregulated in PCa	Khan et al. (2019)
	KDM5C (JARID1C)	H3K4me2/3	Controls the proliferation of prostate cancer cells; might represent a novel therapeutic target.	Stein et al. (2014)
	JMJD2A (KDM2A)	H3K9me3	Promotes prostate cancer initiation. Associate with PCa aggressiveness.	Kim et al. (2016)
		H3K36me3		
	JMJD3 (KDM6B)	H3K27me3	Overexpressed in metastatic prostate cancer	Xiang et al. (2007)
	JMJD6	H3R2me2	Promotes castration resistant disease	Paschalis et al. (2021)
	PADI2	H3R26me	Promotes AR signaling activation and progression of CRPC	Wang et al. (2017)

### 3.2 Manipulating H3 methylation modulators for the treatment of PCa

It seems more evident that PCa initiation and progression, as well as the metastatic potential of prostate tumor cells are characterized by dynamic alterations in the histone (lysine) methylation patterns. A better understanding of the role of epigenetic factors in PCa development and metastasis could lead to new approaches for the prevention, early diagnosis and treatment of this disease. Recent preclinical studies and clinical trials have highlighted the potential of epigenetic therapies that modulate enzymatic activities to complement standard PCa treatments as AR inhibitors (Kumaraswamy et al., 2021).

Only a limited number of molecules that can directly inhibit the active sites of targeted enzymes have been reported. The scientific literature has put forward that KMTs inhibitors, especially EZH2 ones, either alone or in combination with other anti-cancer agents reduce the proliferation of prostate tumors (Upadhyay et al., 2021). Moreover, an inhibitor of LDS1 has recently been described with great activity in stopping tumor growth, being a potential therapeutic agent for hormone-resistant prostate cancer (Etani et al., 2015). As PRMTs have been found to have the ability to regulate cell cycle, could be regarded as a novel potential research target for tumor therapy. PRMTs inhibitors like GMS, MS023, MS049 were found to inhibit some PRMTs (Gong et al., 2020) unfortunately, there is no clinical data available about the role of these ones as therapeutic agents in PCa. Concomitant administration of CI-Amidine (a PADI2 inhibitor) with enzalutamide results in synergistic inhibition of cell proliferation and tumor progression under ADT condition. This treatment strategy may potentially be used to a subset of CRPC patients with PADI2 expression, but further investigations are needed to evaluate whether PADI2 inhibition could be a preventive strategy for PCa progression especially in CRPC patients (Wang et al., 2017).

Other drugs targeting epigenetic factors used in experimental and ongoing clinical trials are summarized in Table 2. Currently, it is obvious that such treatments will be more efficient in combination with standard treatments for which most lethal prostate cancer ultimately develop resistance mechanisms.

**TABLE 2:** Experimental and clinical trials with drug targeting H3 methylation modulators in prostate cancer.

Target	Drug	Combination	Tumor type	Status	Trial number	References
EZH2	CPI-1205	Enzalutamide/Abiraterone or Prednisone	mCRPC	active	NCT03480646	Taplin et al. (2018)
	PF06821 497	SOC	CRPC	recruiting	NCT03460977	Pfizer (2018)
	Tazemetostat (EPZ-6438)	Enzalutamide/Abiraterone or Prednisone	mPCa	recruiting	NCT04179864	Epizyme Inc. (2019a)
	SHR2554	Abiraterone/Prednisone or Ezalutamide	mCRPC	terminated	NCT03741712	Jiangsu HengRui Medicine Co. Ltd. (2018)
	DS3201	Ipilimumab	mCRPC	recruiting	NCT04388852	M.D. Anderson Cancer Center (2020)
WDR5–MLL	OICR- 9429	Cisplatin	PCa	experimental	-	Zhou et al. (2021)
Menin-MLL	MI-503	-	CRPC	experimental	-	Brzezinka et al. (2020)
LSD1	CC-90011	Abiraterone, Prednisone	PCa	Not yet recruiting	NCT04628988	Celgene (2020)
	Phenelzine	Monotherapy/Doxetacel	PCa/CRPC	Completed	NCT01253642	OHSU Knight Cancer Institute (2010)
	SP-2577 (Seclidemstat)	-	Advanced solid tumors	Phase I	NCT03895684	Salarius Pharmaceuticals LLC (2019)
G9a	CM-272	-	2D and 3D *in vitro* models for mCRPC	Not in clinical trial	-	Moreira-Silva et al. (2022)

## 4 Bladder cancer (BCa)

Bladder cancer (BCa) is the 10th most prevalent cancer worldwide, with an increasing tendency, particularly in industrialized and developed countries; it is the most prevalent tumor of the urinary system, with urothelial carcinoma being the prevalent histologic subtype, with a high rate of recurrence and poor outcomes because of relapse (Halaseh et al., 2022). Based on the latest Global Cancer Incidence, Mortality and Prevalence (GLOBOCAN) data, 440,864 new cases of bladder cancer in men were identified in 2020, accounting for around 4.4% of new cancer cases diagnosed in men (Sung et al., 2021; Halaseh et al., 2022). A significant proportion of bladder cancer cases is attributable to tobacco smoking (50%–65%), as well as to occupational, environmental factors (estimated 20% of all cases) and schistosomiasis infection (in Africa and Middle East) (Caballero et al., 2022).

Most cases of bladder cancer are diagnosed as urothelial carcinomas, also known as transitional cell carcinomas (TCCs) and a small percent as squamous cell carcinomas and adenocarcinomas. Most TCCs are non-invasive bladder cancers (NMIBCs, low grade stages carcinoma, Ta and T1), while about a quarter of TCCs are muscle invasive bladder cancer (MIBCs, high grade stages T2-T4) with poor outcomes (Kaufman et al., 2009). The prognosis for NMBC patients is favorable, however more than a half of cases will relapse and a small percentage will progress to MIBC (Li et al., 2016). In this context, understanding the mechanism of BCa development is a challenging objective for cancer management. The newest molecular technologies have uncovered a heterogeneous genetic and epigenetic landscape of BCa origin.

Most of MIBSs display mutations in chromatin remodeling genes, including HDMs, HMTs, SWI/SNF complexes and HATs (Gui et al., 2011; The Cancer Genome Atlas Research Network, 2014). NMIBCs are governed by the dysregulation of cancer stem markers and cell cycle genes expression (The Cancer Genome Atlas Research Network, 2015)*.* Several signaling pathways, e.g., PI3K/mTOR, p53/RB, GFR3 and RAS-MAPK, with a major role in controlling proliferation of urinary bladder cells, are altered due to these mutations (Li et al., 2016).

### 4.1 H3 methylation regulators in BCa

H3K4 methylation is a crucial modification in BCa progression. A decreased level of monomethylated H3K4 was associated with poor prognosis and could be a predictive biomarker for MIBCs (Schneider et al., 2011). A study performed by Wu and collaborators has revealed that the SMYD3 expression level is significantly higher in BCa cell lines than in human normal bladder cells (Wu et al., 2019). WDR5, another enzyme with H3K4 methyltransferase activity was found to be overexpressed in BCa (Chen et al., 2015).

KMT2D (MLL2) is downregulated in BCa related to tumor development, recurrence and resistance to therapy (Meghani et al., 2022). The expression of KMT2C (MLL3) is also altered due to higher grade mutation in NMIBC (Hurst et al., 2017) and in some MIBC subtypes (Robertson et al., 2018). Expression of KDM5 is also altered in MIBC, resulting in cell growth retardation through coregulation of the E2F/RB1 pathway (Meghani et al., 2022).

LSD1 was found to be overexpressed in BCa cells. LSD1 could be a potential target for epigenetic treatment because its inhibition leads to suppression of BCa cells development and proliferation. LSD1 acts via AR and ER pathways (Lan et al., 2013).

In a subset of patients with NMIBC, a global increase of H3K9 and H3K27 methylation associated with high-grade tumors has been reported (Ellinger et al., 2014). Different histone methylation patterns found in MBIC and NMIBC suggest that patients from these subgroups could have different responses to epigenetic therapy. For this reason, there is a stringent need for new biomarkers validation to predict responses to such epigenetic modulators. G9a methyltransferase was found to be overexpressed in BCa, and its inhibition has been shown to affect cell survival via AMPKmTOR pathway. Moreover, an interference between G9a and DNA methyltransferase 1 (DNMT1) has been reported to induce transcriptional silencing of target genes (Tachibana et al., 2008). Additionally, this enzyme may interact with EZH2 allowing the silencing in specific genes, becoming a potential target for advanced metastatic BCa (Martinez et al., 2019).

Many studies have shown that aberrant H3K27 methylation levels occur in multiple cancers, and that altered H3K27me3 levels are correlated with the development of tumors (Ellinger et al., 2014; Santos et al., 2014; Hong et al., 2017). It has been demonstrated that the oncogenic axis Rb-E2F-EZH2 predicts progression and recurrence in NMIBC (Santos et al., 2014). Also, EZH2 promotes the proliferation and migration of BCa cells via the JAK2/STAT3 pathway. EZH2 works by locking tumors in a state of growth causing proliferation and migration of bladder cancer cells, and could be a target for novel treatment strategies in BCa (Chen et al., 2019).

JMJD1A (KDM3A) is overexpressed in BCa and it has been correlated with metabolic reprogramming, resulting in tumor progression (Wan et al., 2017).

Downregulated KDM6A was found with a higher frequency in NMIBC tumors (Meghani et al., 2022). Depletion of KDM6A enhanced anchorage independent growth and cell migration in bladder cancer cell lines). These data have shown that KMD6A plays a role in tumor growth and suppression of cell migration (Ler et al., 2017). Crosstalk between EZH2 and KDM6A has been noted in BCa tumors (Lee and Song, 2017).

Numerous loss-of-function mutations have been found in chromatin modifying genes. For that reason, the corresponding proteins were considered oncogenic drivers in BCa. However, a high frequency of mutations, particularly in MLL2 and KDM6A has also been discovered in nonmalignant urothelium. These findings suggest that additional events are needed to trigger carcinogenesis (Lawson et al., 2020). For example, fibroblast growth factor receptor 3 (FGFR3) activation and KDM6A loss-of-function mutations have been found in MIBC. Such mutations in KDM6A gene promote a permissive environment for FGFR3 activating mutations to drive tumorigenesis in bladder urothelium (Meghani et al., 2022). Another study based on massive exome sequencing using 54 BCa samples revealed that KDM6A and BRCA1-associated protein-1 (BAP-1) mutations co-occur in BCa tumors (Yap et al., 2014). According to all these data, KMD6A is a key player in tumor growth and suppression of cell migration. Moreover, it has been shown that KDM6A loss-of-function mutation could create epigenetic susceptibility to EZH2 inhibitors (Lee and Song, 2017). Also, a high level of KDM7A has been associated with increased proliferative capacity of BCa cells and cisplatin resistance via androgen receptor (Lee et al., 2020).

A limited number of studies have focused on the role of H3K36 methylation in initiation, progression or recurrence of BCa. H3K36 trimethylase NSD1 (KMT3B) and SETD2 (KMT3A) levels have been increased by a NFkB inhibitor, dimethylaminoparthenolide (DMAPT), resulting in cell death (Nakshatri et al., 2015). NSD1 is altered in 6.63% of BCa patients (AACR Project GENIE Consortium, 2017).

A recent study performed by Lu and others (2022) noted a positive correlation between KDM2A gene copy number and its overexpression in BCa (Lu et al., 2022). A KDM2A regulating super-enhancer was discovered in high-grade BCa, resulting in higher expression of KDM2A compared to that in low-grade BCa. In the same study, several key target of KDM2A, including Retinoic Acid Receptor Responder Protein 3 gene (RARRES3) was identified, and the axis KDM2A-RARRES3 could be a potential therapeutic pathway for the high-grade BCa (Lu et al., 2022).

Using bioinformatic tool TIMER2, Qiu and others (2022) highlighted the molecular perspective on the role of CARM1, comparing its expression difference between various cancer types and corresponding normal tissues. In BCa higher expression level of CARM1 was noticed, bringing out its potential value in clinical prognosis (Qiu et al., 2022). PRMT6 knockdown can significantly inhibit the growth of bladder cancer cell lines SW780 and RT4. The number of cells in the S phase were significantly reduces, while those in G0 and G1 phases were increased (Chen J. et al., 2022). These results highlighted the role of PRMT6 in transformation of BCa cells.

Compared to PCa, BCa is less studied regarding H3 methylation regulators. However, the current results are encouraging and highlight the new role for chromatin modifying enzymes in BCa, even if further studies are needed to understand the mechanisms that drive the interference between master regulators (Table 3).

**TABLE 3:** Functional outcomes of H3 methylation master regulators considered as potential therapeutic targets and biomarkers in BCa.

Enzyme type	Enzyme name	PTMs	Function in bladder cancer	References
HMTs	SMYD3	H3K4me2/3	Promotes cell proliferation	Wu et al. (2019)
	WDR5	H3K4me1	Promotes tumor development and proliferation; correlated with advanced tumor stage MBIC and poor survival; decrease in H3K4me1 is correlated with poor prognosis.	Chen et al. (2015)
	KMT2C (MLL3)	H3K4me3	Causes impairments in DNA replication and repair; increased chromatin instability; potential biomarker for high grade NMIBC, and luminal papillary and basal squamous MIBC subtypes.	Rampias et al. (2019)
				Martinez et al. (2019)
	KMT2D (MLL4)	H3K4me1/2	Causes disorders in DNA replication and cell cycle; induced invasion, and migration; recurrence and resistance to therapy	Sun et al. (2019)
				Meghani et al. (2022)
	G9a	H3K9me2	Contributes to the development of NMIBC; possesses immunomodulatory effects	Tachibana et al. (2008)
				Mourits et al. (2021)
	EZH2	H3K27me2/3	Promotes tumor development and progression in BCa; predicts progression and recurrence in NMIBC	Santos et al. (2014)
				Chen et al. (2019)
	NSD1	H3K36me2	Contributes to tumorigenesis	Nakshatri et al. (2015)
KDMs	LSD1	H3K4me1/2/3	Promotes cancer development and proliferation	Lan et al. (2013)
	KDM3A	H3K27me3	Promotes tumor progression	Wan et al. (2017)
	JMJD2A (KDM2A)	H3K9me3 H3K36me3	Promotes higher-grade BCa	Lu et al. (2022)
	KDM6A	H3K27me3	Induces tumor immune escape; activates proinflammatory pathway; induces deregulation in the expression of cell identity related genes.	Kobatake et al. (2020)
				Chen et al. (2021)

### 4.2 Manipulating H3 methylation modulators for the treatment of BCa

The standard clinical approach for NMIBC is trans-urothelial resection followed by chemo- or immune-therapy (Griffiths, 2013). In the case of the aggressive form, MIBC, the standard therapy is cystectomy and, in cases of disease relapse, combinatory drugs containing cisplatin are used. However, the survival rate after treatment is about 1 year, which reflects the stringent needs for the development and validation of new therapeutic agents (Faleiro et al., 2017). Relatively few studies of H3 methylation regulators are currently evaluated in ongoing trials in bladder cancer. Tazemetostat is a drug that targets EZH2. It could be used in combination with other agents to enhance its efficacy, for example, Pembrolizumab (MK-3475), in advanced urothelial carcinoma (NCT03854474). In this case, tazemetostat inhibits EZH2 and stops the growth of tumor cells. Pembrolizumab is a monoclonal antibody that could help the body’s immune system (Piunti et al., 2022). The first developed specific inhibitor of G9a is BIX-01294, which was reported to inhibit tumor cell growth and to induce apoptosis in BCa cells, via ER stress pathway (Kubicek et al., 2007). A natural product named emodin is able to regulate macrophage memory by inhibiting the removal of H3K37me3 marks at the promoter of several key genes (Iwanowycz et al., 2016). To date, the development of inhibitors of HMTs and HDMs for BCa has been still in a primordial stage (Table 4). However, even if the epidrugs are still in the beginning as a therapeutic class of molecules, the combination with traditional therapies is a feasible approach to improve the effectiveness of classical drugs, is useful in overcoming resistance, and even in reducing toxicities and secondary effects.

**TABLE 4:** Experimental and clinical trials with drug targeting histone methylation modulators in BCa cancer.

Target	Drug	Combination	Tumor type	Status	Trial number	References
PRMT5	GSK3326595	Pembrolizumab	Metastatic transitional cell carcinoma of the urinary system (mTCC)	active	NCT02783300	GlaxoSmithKline (2016)
	PF-06939999	-	Bladder carcinoma	active	NCT03854227	Pfizer (2019)
EZH2	Tazemetostat	Pembrolizumab	Stage III-IV BCa Metastatic Urothelial Carcinoma	active	NCT03854474	National Cancer Institute (2019)
	CPI-0209	-	Urothelial carcinoma (solid tumors)	active	NCT04104776	U.S. National Library of Medicine (2019)
G9a	BIX-01294	-	Urothelial carcinoma	Not in clinical trial	-	Cui et al. (2015)
	CM-272	-	Urothelial carcinoma	Not in clinical trial	-	Zhu et al. (2019)

## 5 Renal cancer (RCa)

Renal cancer has one extremely prevalent subtype, namely, renal cell carcinoma (RCC), which accounts for 90% of the cases. This subtype has its origin in renal tubular cells and it can be further classified into multiple histopathological subtypes, such as clear cell RCC (ccRCC) which is the most frequent, papillary RCC (pRCC), sarcomatoid RCC (sRCC) and chromophobe RCC (Moch, 2013). A third of RCC patients ultimately die from the disease, which makes RCC the deadliest type of genitourinary cancer.

Several etiologies of RCC have been identified, each subtype having characteristic risk factors. Notably, several disorders have been associated with RCC. For instance, the chromophobe subtype is associated with the Birt-Hogg-Dube syndrome, while type 2 ppRCC is associated with hereditary leiomyomatosis. Type 1 pRCC patients have germline mutations in the MET oncogene and ccRCC patients usually have loss of function VHL, either through mutations or promoter methylation. VHL is a tumor suppressor and is involved in the VHL-HIF pathway, its loss leading to an overexpression of HIF1 and HIF2, which also leads to VEGF overexpression triggering events such as proliferation, apoptosis and angiogenesis. Gene mutations commonly identified in other cancers such as RAS, tp53 and PTEN are not usually found in RCC. Furthermore, a plethora of microRNAs have been studied in relation to RCC and could be manipulated for treatment (Joosten et al., 2018). Data on histone methylation, however, is quite scarce. Given its close relationship with DNA methylation, which may play a role both in initiation and in treatment of RCC subtypes, this topic could be worth further investigation.

### 5.1 H3 methylation regulators in RCa

In RCC, methylation patterns could be disturbed by mutations in the SETD2, KDM5C and KDM6A genes. However, these methylation patterns have not been extensively investigated. Genome-wide sequencing studies have identified mutations in KDM5C, KDM6A, SETD2 in ccRCC, most of them being loss of function mutations.

One study performed on tissue microarray using immunohistochemistry found intense staining of H3K4me3 in ppRCC and relatively similar intensities of H3K4me1 and H3K4me2 among the RCC subtypes (Ellinger et al., 2010). Interestingly, the study identified the H3K4me score as a predictor of progression-free survival for RCC patients, suggesting the role of H3K4 methylation as a prognosis marker. Specifically, patients with lower levels of H3K4me1/me2/me3 had a poor prognostic, which is in accordance with another study by Seligson and collaborators, (2009). The latter looked at H3K4me2 levels, as well as H3K9me2 levels and found a correlation between low levels of both modifications and poor patient outcomes (Seligson et al., 2009). Additionally, Rogenhofer and collaborators found more intense staining of H3K27me1/2/3 in pRCC than in ccRCC. Concerning H3K36me3, loss of function mutations in SETD2 were found in 10% of RCCs leading to decrease in H3K36me3 (Rogenhofer et al., 2012). Consequently, genome stability is affected and global DNA methylation is decreased (Angulo et al., 2021). Lastly, H3K79 has not been studied in relation to RCa, but higher expression of DOT1L has been associated with poor clinical outcomes in ccRCC patients (Hong et al., 2020).

SMYD2 was found to regulate the expression of miR-125b, which in turn regulates the Wnt/B-catenin pathway, acting as a tumor suppressor. Activation of the SMYD2/miRNA pathway apparently inhibits sunitinib, weakening its effects and accelerating tumor growth (Yan et al., 2019). Sunitinib is a therapeutic drug belonging to the class of tyrosine kinase inhibitors (TKIs), which are regarded as the first-line treatment of RCC. EZH2 expression was also linked to sunitinib resistance in RCC and could even have a prognostic role (Eichenauer et al., 2021; Li et al., 2021). G9a, however, has been suggested as a potential target for ccRCC treatment, as it can inactivate tumor suppressors in the event of hypoxia, which is also correlated to the pathway mentioned above (Hong et al., 2020). Furthermore, G9a was recently found to downregulate SPINK5 through its methyl-transferase activity. Inhibition of G9a leads in turn to inhibition of proliferation, migration and invasion of RCa, and thus could be a promising therapeutic target (Li et al., 2021). Potential therapeutic targets are summarized in Table 5.

**TABLE 5:** H3 methylation regulators considered as potential therapeutic targets in RCa.

Enzyme type	Enzyme name	PTMs	Function in RCa	References
HMTs	SMYD2	H3K4me, H3K36me2	Involved in the SMYD2/miRNA pathway; its inhibition acts as a tumor suppressor	Yan et al. (2019)
	G9a	H3K9me2	Associated with tumor proliferation, migration and invasion	Li R. G. et al. (2021)
	EZH2	H3K27me2/3	Overexpressed in renal tumor cells, promotes development and metastasis of RCC	Tingting Li et al. (2021)
	SETD2	H3K36me3	Associated with genome stability and DNA methylation in RCC	Rogenhofer et al. (2012)
				Angulo et al. (2021)
	DOT1L	H3K79me2/3	Associated with poor clinical outcomes in ccRCC	Qu et al. (2016)
HDMs	JMJD1A (JHMD2A)	H3K9me1/2	Overexpressed in RCC	Henrique et al. (2012)
		H3K36me2/3		
	JMJD2B (KDM4B)	H3K27me3	Overexpressed in RCC	Henrique et al. (2012)
		H3K36me3		
	JMJD6	H3R2me2	Increases tumor sensitivity to sunitinib	Zhang et al. (2021)

HIF1 is known to induce JMJD1A/B and as such these HDMs could play an indirect role in ccRCC where HIF is upregulated. A couple of studies found elevated levels of JMJD1A in RCC cell lines and cancer tissues, one of which also found an elevated level of JMJD2B (Henrique et al., 2012). Additionally, JMJD6 has been studied in relation to sunitinib sensibility, results showing that JMJD6 inhibitors act synergistically with sunitinib, increasing tumor sensitivity to it (Zhang et al., 2021).

Of the arginine methylases, PRMT1 and PRMT5 have been suggested as important players in kidney development and maintenance. Thus, their relationship with RCa could be worth investigating. PRMT1 has already been studied in ccRCCs, results showing higher expression in low grade and low stage of ccRCC and loss of PRMT1 in high grade and high stage ccRCC, showcasing its duality (Filipović et al., 2019). Moreso, another study involved the inhibition of PRMT1, which lead to increased sunitinib sensitivity and tumor growth inhibition (Wang et al., 2021). However, PRMT1 is not involved in the methylation of H3 arginine residues, but on H4 (H4R3me2a). PRMT5 symmetrically demethylates H3R8, as well as the residue mentioned above, and as such there could be crosstalk between the two (Guccione and Richard, 2019).

### 5.2 Manipulating H3 methylation modulators for the treatment of RCa

The main therapeutic approaches for RCa are surgery and several TKIs, particularly monotherapy with sunitinib. However, TKI resistance is not uncommon and also involves epigenetic alterations, which pushes the need for the investigation of epigenetic drugs. Recent advances have led to the approval of immune checkpoint inhibitors (ICIs) as adjuvants by the FDA and EMA, for instance pembrolizumab. Still, as previously described, resistance to adjuvants can develop and has dramatic consequences.

Given that few studies have focused on H3 methylation regulators in RCa, not much is known about their roles in this cancer type and, consequently, the modulators have not been extensively studied either. So far, inhibitors of EZH2 seem to have the greatest potential of being used in adjuvant therapy, as EZH2 has been associated with tumor growth, metastasis and angiogenesis in several studies both *in vivo* and *in vitro* (Li R. G. et al., 2021). Moreso, EZH2 has been investigated in relation with sunitinib resistance in mice, the results warranting further studies (Adelaiye-Ogala et al., 2017). A very recent study on ccRCC cell lines showed that inhibition of EZH2 induces anticancer effects through increased apoptosis, reduced invasive and wound healing capacities (Hong et al., 2023). The EZH2 inhibitor used was tazemetostat, which is currently in clinical trials for RCa treatment, among other cancer types (Table 6). Although these clinical trials are not limited to studies on RCa, but include other types of tumors, they can provide valuable new information on tazemetostat’s potential. Another clinical trial which includes patients with RCa is testing the combination of another EZH2 inhibitor, namely, valemetostat, and an ICI, ipilimumab (clinicaltrials.gov/ct2/show/NCT04388852).

**TABLE 6:** Experimental and clinical trials with drug targeting H3 methylation modulators in RCa.

Target	Drug	Combination	Tumor type	Status	Trial number	References
EZH2	Tazemetostat	-	RCC	Expanded access	NCT03874455	Epizyme Inc. (2019b)
	Tazemetostat	-	Renal Medullary Carcinoma Rhabdoid Tumors of the Kidney	Phase 2	NCT02601950	Epizyme Inc. (2015)
	Valemetostat (DS3201)	Ipilimumab	mCRCC	Recruiting	NCT04388852	M.D. Anderson Cancer Center (2020)
G9a	BIX-01294	TRAIL	Human RCa cell lines	Experimental	-	Woo et al. (2018)

While there are currently no ongoing clinical trials focusing solely on RCa, several targets have been identified and could thus constitute great starting points for an epigenetic-based therapy that mitigates the risk of chemoresistance.

## 6 Testicular cancer (TCa)

Albeit not as prevalent and aggressive as the previously described cancers, testicular cancer represents a major cause of death for men in the 15–45 years age group, being the most diagnosed cancer in this particular group (Ghazarian et al., 2017; Gurney et al., 2019). TCa refers to testicular germ cell tumors (TGCT), sex cord-gonadal stromal tumors and secondary testicular tumors (Boccellino et al., 2017). TGCTs are the most common subtype, covering 90%–95% of all TCa cases, and they also have three subtypes of their own: seminomatous germ cell tumors (SGCTs), non-seminomatous germ cell tumors (NSGCTs) and spermatocytic tumors (Ghazarian et al., 2015; Amin et al., 2017). SGCTs and NSGTs share the same origin, known as germ cell neoplasia *in situ*, which is a precursor lesion that arises during embryonic development and possibly explains the similarities between SGCTs and primordial germ cells (PGCs). NSGCTs can have extra-embryonic tissues and their subtypes are more aggressive. These subtypes are, namely, embryonal carcinomas, yolk sac tumors, choriocarcinomas and teratomas. Mixed tumors containing both SGCT and NSGCT elements are also commonly diagnosed and included in NSGCTs (Amin et al., 2017; Martinot et al., 2018). Despite their common origin, SGCTs and NSGCTs have distinct molecular characteristics which translates into a need for different diagnostic and therapeutic approaches (Umbreit et al., 2020).

Not much is known about the etiology of TGCTs, as there are currently no strong candidates considered responsible for its induction. However, a series of risk factors have been identified, and the likely explanation for the occurrence of TGCT could be the interplay between these risk factors, both genetic and environmental (Baroni et al., 2019). On the genetic side, there is a correlation between TGCT and mutations in genes such as KITLG and KRAS, as well as risk loci that contain genes responsible for DNA damage repair, microtubule assembly and telomerase function (Litchfield et al., 2015; Ding et al., 2019; de Vries et al., 2020). Similar to PCa, the AR has been associated with TGCT, given the possible involvement of hormone levels in TGCT development, but the data so far has been inconsistent (Martinot et al., 2018). Endocrine disruptors (EDs) can sometimes bind to AR and alter its localization or function, which is the case of Bisphenol A (BPA) (Landero-Huerta et al., 2017; Lombó and Herráez, 2021). EDs are environmental factors that could contribute to TGCT initiation and development by exposure during pregnancy, so far studies suggesting an indirect role. Other risk factors include cryptorchidism, hypospadias, high maternal age, premature birth which are sometimes associated with exposure to EDs as well (Vega et al., 2012; Singh et al., 2021). Environmental factors have also been associated with alterations of the epigenetic code of cells, which led to investigating the involvement of epigenetic mechanisms in TGCT initiation, progression, and even treatment. As epigenetic reprogramming plays a major role in male germline development, the field of epigenetics is of particular interest for TGCT and as such, many studies now focus on it and acknowledge its contribution as a major player (Oakes et al., 2007; Ghazarian et al., 2015; Singh et al., 2021). So far, most of the data comes from research focused on DNA methylation, histone acetylation and, more recently, various types of microRNAs, while histone methylation remains understudied. The information on H3 methylation at various residues is presented in what follows in association with TGCT, whereas other epigenetic modifications are described in several reviews (Singh et al., 2021; Nicu et al., 2022).

### 6.1 H3 methylation regulators in TCa

Compared to PCa, the information on H3 methylation is quite scarce in association with TGCT. Although the past decade has seen an increase in studies regarding the epigenetic code of germ cells and the role of epigenetic mechanisms in spermatogenesis, this interest has not yet translated to similar studies on TGCT. One hurdle could be the heterogeneity of tumors which makes it hard to study particular modifications in a controlled setting. Hence, data regarding histone modifications is currently limited, but exploring the various enzymes involved in their regulation could prove fruitful from a therapeutic point of view (Table 7).

**TABLE 7:** H3 methylation regulators that could serve as potential therapeutic targets in TCa.

Enzyme type	Enzyme name	PTMs	Function in TCa	References
HMTs	SMYD3	H3K4me2/3	Upregulated in TGCT cell lines	Lambrot and Kimmins (2011)
	G9a	H3K9me2	May promote tumor growth Overexpressed in NSGT	Ueda et al. (2014)
				Lobo et al. (2018)
	EZH2	H3K27me2/3	Underexpressed in TGCT; overexpressed in GCNIS and NSGT; absent in EC	Hinz et al. (2010)
				Almstrup et al. (2010)
				Lambrot and Kimmins (2011)
				Lobo et al. (2018)
KDMs	LSD1	H3K4me1/2/3	Expressed in all TGCT subtypes; upregulated in SGCT; when inhibited, interferes with tumor growth	Wang et al. (2011)
				Lourenço et al. (2022)

Methylation of H3K4, particularly the H3K4me3 mark, has a stage-specific distribution in normal testis, but not in TGCTs (Vega et al., 2012; Burlibaşa et al., 2021). High levels of H3K4 methylation are present in GCNIS, as well as in non-seminoma, which, according to Singh et al. (2021), could be related to the proliferative capacity by preventing DNA methylation, which is necessary for differentiation (Singh et al., 2021). At a more specific level, H3K4me3 was identified distally to transcription start sites in a study involving TGCT cell lines by van der Zwan and collaborators (2014). The cell lines, TCam-2 which serves as a SGCT model, and NCCIT, which serves as an embryonal carcinoma model, were characterized through sequencing, methylation microarrays and ChIP-seq. Thus, H3K4me3 was placed in a wider landscape, with the results indicating the similarity between TCam-2 cells and germ cells, in contrast to NCCIT which was more stem cell-like. Mainly, H3K4me3 was enriched at SOX17 in the SGCT cell line and at SOX2 in the NCCIT cell line, which corresponds to their known patterns in SGCT and embryonal carcinoma, respectively (van der Zwan et al., 2014). H3K4 methylases were studied *in vitro* in relation with TGCT only in cell lines, with an upregulation of the SMYD3 gene being noticed by Lambrot and Kimmins (2011). In normal testis, SMYD3 expression is low in fetal tissues, but high in adults (Bernard et al., 2021). One demethylase on the other hand, namely, LSD1, was studied on tumor assays and TGCT cell lines. One study found an upregulation in SGCTs, and another study also found an upregulation in the stem cell lines analyzed (Wang et al., 2011; Yin et al., 2014). Additionally, the first study suggests that an inhibition of LSD1 can interfere with tumor growth (Wang et al., 2011). Moreover, a recent retrospective study looked at LSD1 expression in a cohort of patients, identifying LSD1 expression in all the histological subtypes of TGCT. While this could seem discouraging due to a lack of histological specificity, inhibiting LSD1 could prove to be a viable option for all TGCT patients in the future (Lourenço et al., 2022).

Methylation of H3K9 was similarly detected in all cellular stages of non-seminoma, while low levels of H3K9me2 were detected in GCNIS (Almstrup et al., 2010; Vega et al., 2012). The enzymes targeting H3K9, the methylase G9a and demethylase JMJD1A were investigated by Ueda et al. (2014), on cell lines, animal models and tumor assays. The loss of the mentioned enzymes had antagonistic effects, with the loss of G9a leading to a decrease in tumor growth, and the loss of JMJD1A leading to an increase in tumor growth. Moreover, JMJD1A protein levels were lower in both SGCTs and NSGCTs compared to normal testis (Ueda et al., 2014).

As for enzymes targeting H3K27, a couple of studies concerning EZH2 date back to 2010. One study performed on frozen tissue found a decrease in EZH2 levels in TGCT compared to normal testis, with no significant differences between SGCTs and NSGTs, while the other found high levels of EZH2 in GCNIS and reported its absence in embryonal carcinoma (Almstrup et al., 2010; Hinz et al., 2010). The latter study also found that the H3K27 demethylases JMJD3 and KDM6A could not be identified in the formalin fixed paraffin embedded samples analyzed through IHC (Almstrup et al., 2010). In normal spermatogenesis, EZH2 is localized in round spermatids and plays a role in the extensive chromatin remodeling that takes place. The low level of this enzyme in TGCTs compared to normal testis could therefore be involved in an aberrant spermatogenesis process in TGCTs (Lambrot and Kimmins., 2011).

An *in silico* analysis found that KMT2B/C/D, KDM4D and KDM3A, are overexpressed in SGCTs. As these enzymes either methylate the H3K4 residue, or demethylate the H3K9 residue, they are mainly associated with the establishment of activation marks. Conversely, G9a and EZH2, which are associated with repressive marks, are overexpressed in NSGTs (Lobo et al., 2018). While this is only an *in silico* model, such studies where multiple key players in epigenetic regulation are investigated simultaneously could provide invaluable insights into the biology of TGCTs, accelerating the discovery of potential treatment adjuvants.

Although methylation of H3K79 has not been studied yet in TGCT, it may prove to be of interest. A recent study on the H3K79 methyltransferase, DOT1L, has shown that it is responsible for the self-renewal of germline stem cells in mice, its loss leading to Sertoli cell only syndrome (Lin et al., 2022). While the implications in TGCT are currently unknown, investigating DOT1L may yield interesting information on alternative therapeutic approaches.

Similarly, H3R methylation has not been studied in relation to TGCT, however PRMT5 seems to be involved in PGC specification in mice (Müller et al., 2021). Given its involvement in maintaining the unipotent cell linage, and the direct association between PGC and TGCT origin, further investigations into PRMT5’s role could prove to be valuable.

### 6.2 Manipulating H3 methylation modulators for the treatment of TGCT

Current treatment options for TGCT include orchiectomy, radiation therapy and chemotherapy (Oing et al., 2018). Although the survivability rate is high (90%–95%), chemoresistance occurs in 10%–15% of tumors leading to a dramatic prognosis and poor quality of life (Liao et al., 2018; Groot et al., 2020; Hellesnes et al., 2021). Furthermore, the chemotherapy agents used, bleomycin, cisplatin and etoposide have been associated with epigenetic processes, which call for further studies concerning the consequences of treatment, as well as the possible identification of new therapeutic agents. Epigenetic drugs could prove to be an important addition to the arsenal, but so far information is limited to HDAC and DNMT inhibitors (Nicu et al., 2022).

Thus far, few studies have focused on the potential of modulating HMTs and HDMs for the treatment of TGCTs. Some pre-clinical studies have looked at HDMs inhibitors, specifically of LSD1. One such study found that by inhibiting LSD1 with a CBB compound, the growth of TGCT cell lines was blocked (Wang et al., 2011). A more recent study involved another small inhibitor of LSD1 and the usage of mouse cell lines, yielding a similar result in which embryonic carcinoma cells were inhibited. Furthermore, the authors showed a downregulation of SOX2 and OCT4 proteins, which are involved in pluripotency and associated with TGCT (Hoang et al., 2018). Another group looked at EZH2 inhibitors, which they consider inappropriate in combination with cisplatin, as cisplatin-resistant tumors may already have reduced EZH2 levels (Samaržija et al., 2022).

More recently, a histone methyltransferase inhibitor and a histone demethylase inhibitor, namely, Chaetocin and JIB-04, were used alongside other epigenetic inhibitors to decrease the cell viability in TGCT cell lines (Müller et al., 2022). The results are promising, showing an efficacy in decreasing viability even for cisplatin-resistant TGCTs. However, the authors identified off-target effects beyond the expected histone modifications and characterized them. While Chaetocin is a SUV39H1 inhibitor, off-target modifications included an upregulation of H3K4me2/3 and downregulation of H3K36me2. Similarly, off-target effects of JIB-04, which inhibits KDM5A, included an upregulation of H3K9me3 and downregulation of H3K27me2 and H3K79me3, among others. Other epigenetic inhibitors, such as PRC inhibitors, also induced alterations in the histone methylation landscape, as chromatin remodeling and histone modifications are intimately associated (Müller et al., 2022). Despite these various possibilities, there are currently no ongoing clinical trials involving methylation modulators in TCa (clinical.trials.gov). Extensive research is needed in order to fill in the knowledge gaps, which would undoubtedly lead to a better understanding of the mechanisms and enzymes described and could reveal novel therapeutic strategies.

## 7 Future directions for the development of specific biomarkers and tailoring the epigenome for the treatment of urogenital cancers

**FIGURE 3:**
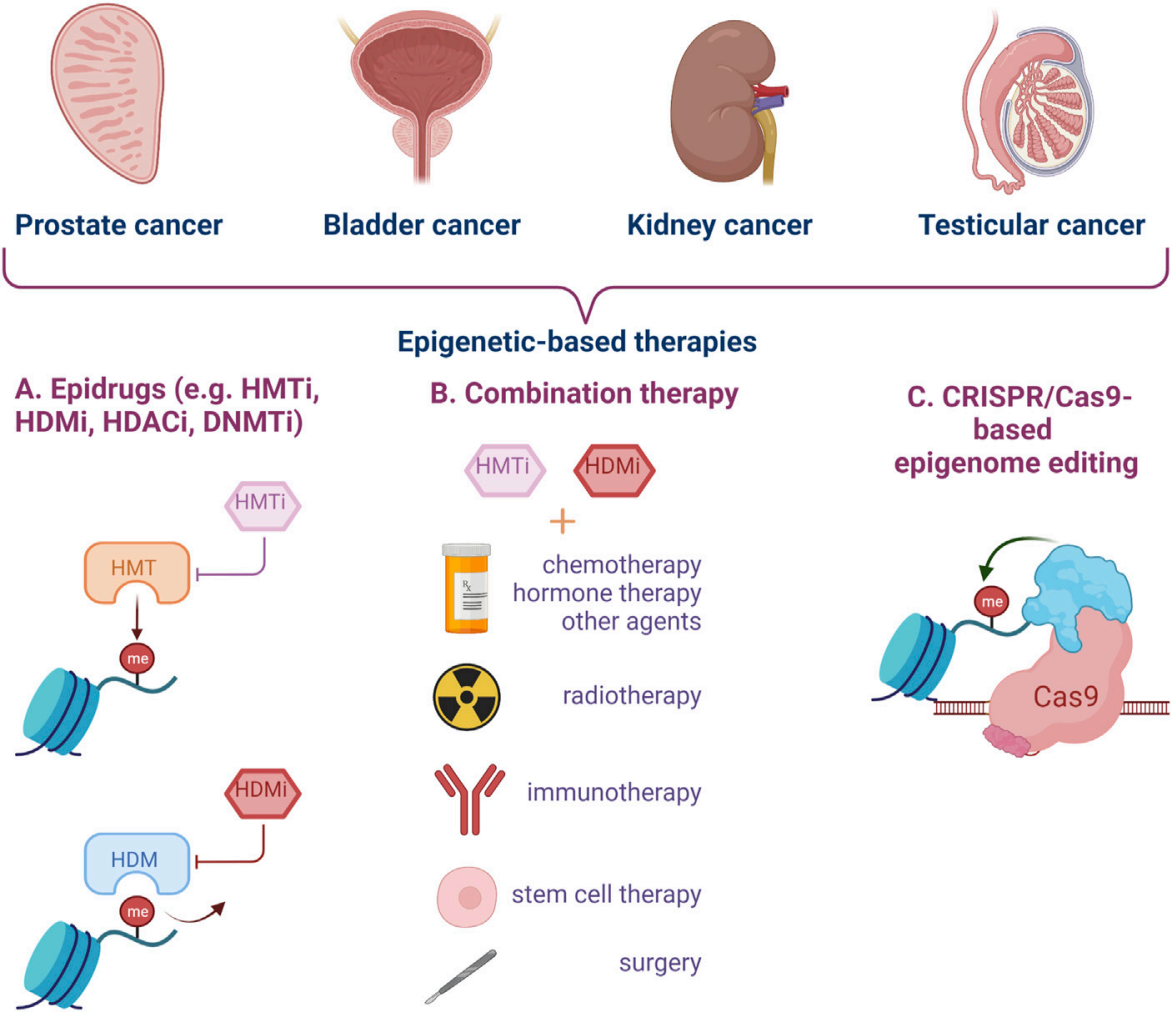
Epigenetic targeting strategies for genitourinary cancer. Epidrugs can be used alone or in combination with other types of treatment to improve the clinical management of aggressive cancers, disease relapse and resistance to classical therapy. Epigenome editing by CRISPR-Cas9 technology represents a new and modern approach for urogenital cancers. (HMTi–Histone-methyl-transferase inhibitors, HDMi–Histone-demethylase inhibitors, HDACi–Histone-deacetylase inhibitors, DNMTi–DNA methyltransferase inhibitors) (Created with BioRender.com).

New strategies and tools need to be developed for early diagnosis and treatment of urogenital cancers, in particular to enhance current pharmacological results (Figure 3).

One such new strategy could be provided by the study of bivalent marks, which are becoming increasingly recognized as important players in cell differentiation, both normal and malignant. The term of bivalent marks refers to the simultaneous presence of antagonistic markers H3K4me3 and H3K27me3 at a certain locus, commonly a key differentiation gene. This occurs during embryonic development in order to maintain pluripotency, by preventing the silencing of genes at the same time as maintaining their low expression. One of the markers is lost when the cell differentiates, so that the poised gene is either activated or silenced. A newer concept however is that of DNA methylation prevention, instead of poising genes for rapid activation (Kumar et al., 2021). Given their implications in pluripotency, bivalent marks could be of particular interest in TGCTs. So far, no *in vitro* or *in vivo* studies have been conducted on this matter, but one *in silico* study managed to identify bivalent marks and their regulators as potential therapeutic targets (Alarcón et al., 2021). Thus, in the near future, mapping of these loci, especially of bivalent marks, along with *in silico* studies could be a predictive tool for tumor staging and cancer treatment used in order to combine different epigenetic drugs.

ChIP-on-chip data and microarray gene expression studies performed by Ke and collaborators have revealed that H3K4me3 and H3K27me3 are associated with differential gene expressions in PCa and primary cells, and the simultaneous presence of the two antagonistic markers at the promoters of some regulatory gene modules represent a particular epigenetic pattern (Ke et al., 2009). The imbalance of switches between H3K4me3 and H3K27me3 leads to epigenetic disturbances of oncogenes and tumor suppressor genes, which have been found to be over- and respectively underexpressed in PCa cells. Bivalent chromatin (H3K4me3/H3K27me3) represents an epigenetic signature of proliferative cells, and therefore could be a potential biomarker for the diagnosis, but especially for the prognosis of prostate cancer.

Tailoring the epigenomic state of cancer cells represents a new tool for personalized medicine. Some studies have focused on epigenetic editing which constitutes a promising strategy, not only to elucidate the mechanistic involvement in stimulating oncogenic processes, but also for therapeutic management. In this context, CRISPR- Cas9 gene activation system was used in histone methylation regulation. PRMD9, SMYD3 and DOT1L, three HMTs were fused with dCas9 in order to obtain a more potent and long-lasting epigenetic modulation for prostate cancer (Kim et al., 2015; Cano-Rodriguez et al., 2016; Pacheco et al., 2022). Specific mutations in MLL into BCa cells were introduced by CRISPR-CAS9 method. These mutated cells exhibited enhanced H3K4me3 modifications, elevated GATA4 and ETS1 expression, which confer resistance to epirubicin therapy and represent a model for studying mechanisms of drug resistance in bladder cancer cells. Moreover, MLL mutations, overexpressed GATA4 and ETS1 could be potential biomarkers for diagnosis and targets for treatment of BCa relapse (Wu et al., 2016). An interesting CRISPR-dCAS9-based system was constructed by Huang and his collaborators. They have developed a light-inducible genetic circuit that only activates the target gene expression by blue light, named Split CRISPR-dCAS-Based Light-Inducible System (Huang et al., 2021). The authors have investigated the potential therapeutic use of this system in BCa cell lines by altering the expression of p53 and E-cadherin protein genes. This work represents an innovative system and a potential strategy for precise inhibition of BCa cells proliferation and invasion (Huang et al., 2021).

## 8 Conclusion

Despite the high number of studies showcasing the critical role of epigenetic deregulation in the pathogenesis of genitourinary cancers, the need for further research becomes obvious when you delve into the topic, with various areas of interest where information is insufficient or unclear being highlighted in this review. Deciphering these epigenetic signatures could help differentiate between cancer subtypes and even predict the patient outcome. Expanding our knowledge on the epigenetic pathways involved in the initiation and progression of genitourinary malignancies could contribute to an early and accurate diagnosis which accounts for disease particularities. Additionally, understanding the epigenetic state of cancer cells could facilitate its modulation and ultimately contribute to the development of epigenetic-based targeted therapy, thus leading to a well-rounded approach which serves to improve patient care.
